# Next-Generation Sustainable Composites with Flax Fibre and Biobased Vitrimer Epoxy Polymer Matrix

**DOI:** 10.3390/polym17141891

**Published:** 2025-07-08

**Authors:** Hoang Thanh Tuyen Tran, Johannes Baur, Racim Radjef, Mostafa Nikzad, Robert Bjekovic, Stefan Carosella, Peter Middendorf, Bronwyn Fox

**Affiliations:** 1 Department of Mechanical Engineering and Product Design Engineering, Swinburne University of Technology, Melbourne 3122, Australia; rradjef@swin.edu.au (R.R.); mnikzad@swin.edu.au (M.N.); 2 Institute of Aircraft Design, University of Stuttgart, 70569 Stuttgart, Germany; baur@ifb.uni-stuttgart.de (J.B.); carosella@ifb.uni-stuttgart.de (S.C.); middendorf@ifb.uni-stuttgart.de (P.M.); 3 Faculty of Mechanical Engineering, University of Applied Sciences Ravensburg-Weingarten, 88250 Weingarten, Germany; robert.bjekovic@rwu.de; 4 Deputy Vice-Chancellor (Research and Enterprise), University of New South Wales, Sydney 2052, Australia

**Keywords:** biopolymer, recyclable thermosets, resin infusion, natural fibre-reinforced composite, sustainability

## Abstract

This work presents the development of two vanillin-based vitrimer epoxy flax fibre-reinforced composites, with both the VER1-1-FFRC (a vitrimer-to-epoxy ratio of 1:1) and VER1-2-FFRC (a vitrimer-to-epoxy ratio of 1:2), via a vacuum-assisted resin infusion. The thermal and mechanical properties of the resulting vitrimer epoxy flax composites were characterised using thermal gravimetric analysis (TGA), differential scanning calorimetry (DSC), dynamic mechanical analysis (DMA), and mechanical four-point bending tests, alongside studies of solvent resistance and chemical recyclability. Both the VER1-1-FFRC (degradation temperature T_deg_ of 377.0 °C) and VER1-2-FFRC (T_deg_ of 395.9 °C) exhibited relatively high thermal stability, which is comparable to the reference ER-FFRC (T_deg_ of 396.7 °C). The VER1-1-FFRC, VER1-2-FFRC, and ER-FFRC demonstrated glass transition temperatures T_g_ of 54.1 °C, 68.8 °C, and 83.4 °C, respectively. The low T_g_ of the vitrimer composite is due to the low crosslink density in the vitrimer epoxy resin. Particularly, the crosslinked density of the VER1-1-FFRC was measured to be 319.5 mol·m^−3^, which is lower than that obtained from the VER1-2-FFRC (434.7 mol·m^−3^) and ER-FFRC (442.9 mol·m^−3^). Furthermore, the mechanical properties of these composites are also affected by the low crosslink density. Indeed, the flexural strength of the VER1-1-FFRC was found to be 76.7 MPa, which was significantly lower than the VER1-2-FFRC (116.2 MPa) and the ER-FFRC (138.3 MPa). Despite their lower thermal and mechanical performance, these vitrimer composites offer promising recyclability and contribute to advancing sustainable composite materials.

## 1. Introduction

The current demand for traditional epoxy composites in industries such as automotive, aerospace, and others is experiencing significant growth, driven by their exceptional performance characteristics such as dimensional stability, thermal stability, and high resistance to harsh conditions (heat, chemicals, water, moisture, etc.). [1,2]. Furthermore, epoxy composites are prized for their high strength-to-weight ratio, durability, and excellent adhesive properties, making them ideal for use in applications where performance and reliability are paramount [2,3]. Indeed, the production of epoxy composite is estimated to reach at least 4 million tons by 2030 [3]. However, the production of epoxy resin is heavily dependent on petroleum resources. Moreover, the recyclability of epoxy composites is limited due to their permanent crosslinked networks, which consequently contributes to significant waste generation and environmental pollution. It is predicted that epoxy carbon fibre-reinforced composite (ER-CFRC) waste will reach up to 263,000 tonnes by 2030 [4].

Incorporating natural fibres into traditional epoxy composites has emerged as a more sustainable and eco-friendlier alternative. Natural fibres, such as jute, hemp, flax, and sisal, are being increasingly explored as reinforcement materials in epoxy composites because they are renewable, biodegradable, and cost-effective [5]. Indeed, the cost for glass and carbon fibre composites is approximately 1200–1800 USD/ton and 12,500 USD/ton, respectively. In contrast, natural fibre composites are much more affordable, costing between 200 and 1000 USD/ton [6]. Moreover, these natural fibre-reinforced composites (NFRCs) show great potential across various industries, including automotive, construction, packaging, and consumer goods, offering a more environmentally friendly solution without compromising performance [6,7]. Moreover, while natural fibres have lower mechanical performance compared to carbon fibres, their performance might be close to glass fibres, thus potentially making them viable substitutes in certain applications.

Moreover, in response to the growing emphasis on decarbonisation and the circular economy, research into biobased vitrimer matrices and their application in natural fibre-reinforced composites has gained significant attention. For example, various vitrimer matrices have been synthesised from a wide range of renewable resources such as tung oil [8], vanillin [9,10], tannic acid and boric acid [11], and rosin [12], among others. Vitrimers are a new class of polymer with dynamic covalent bond networks that combine the advantages of both thermosets and thermoplastics, allowing for better recyclability and reprocessing without compromising the material’s structural integrity [13]. By leveraging the renewable nature of natural fibres, the recyclability of vitrimer chemistry, and the potential for biobased resins, biobased vitrimer epoxy natural fibre-reinforced composites (VER-NFRCs) offer significant environmental benefits in terms of reducing waste, lowering energy consumption, and decreasing the reliance on petroleum resources.

Recent studies [6,14,15,16,17] have explored the integration of bio-based vitrimer epoxy resins into natural fibre composites to enhance their sustainability. For instance, Monteserin et al. [6] employed a vanillin-based vitrimer epoxy as a polymer matrix for the production of flax fibre composites. The resulting composite exhibited a glass transition temperature (T_g_) in the range of 45.8–88.5 °C. In another study, Debsharma et al. [14] synthesised a resorcinol-based epoxy vitrimer resin and employed the vacuum-assisted resin infusion (VARI) technique to fabricate a flax fibre composite. The obtained biobased composites demonstrated a glass transition temperature of 59 °C and were also successfully reshaped when held at 190 °C for 20 min, indicating potential for material reusability. Fei et al. [15] investigated a hemp fibre-reinforced composite with a dual dynamic network vitrimer, using a bio-based matrix derived from hempseed oil and limonene derivatives. The matrix was then used to make hemp fibre reinforced composites exhibiting a T_g_ between 72.6 and 73.9 °C, with tensile strengths and moduli being observed at approximately 50 MPa and 3500 MPa, respectively. In addition, Rohewal et al. [17] developed a fatty acid-based vitrimer composite with flax fibres, which showed a T_g_ ranging from 32.8 °C to 43.1 °C, a lower value compared to other biobased vitrimer flax composites reported.

While the potential of VER-NFRCs is considerable, several challenges still hinder their widespread use in industrial applications. Beyond the inherent moisture sensitivity of natural fibres, one of the primary barriers is the high viscosity of vitrimer resins [18,19]. This rheological limitation restricts processability and makes common industrial-scale manufacturing methods, such as resin infusion and resin transfer moulding (RTM), less feasible [20,21]. As a result, production is currently limited to techniques like prepreg and stamp forming, which are less efficient for high-volume manufacturing [21]. Addressing these viscosity-related challenges is crucial for expanding the industrial viability of vitrimer-based natural fibre composites.

In this work, a catalyst-free, biobased vitrimer hardener was created by reacting vanillin with an industrial-grade amine hardener (Aradur^®^ 3487) through an imine condensation reaction. This vanillin-based vitrimer hardener was then cured with an industrial-grade epoxy resin (Araldite^®^ LY 1564), resulting in a vanillin-based vitrimer epoxy resin (VER). The VER was used as a polymer matrix to fabricate a vitrimer epoxy flax composite (VER-FFRC) via the VAP^®^ process. The thermal and mechanical properties of the VER-FFRC were evaluated and compared to those of conventional epoxy flax composites (ER-FFRCs) through a variety of tests, including DSC, TGA, DMA, and flexural four-point bending tests. The solvent resistance and chemical recyclability of the VER-FFRC were also evaluated. The objective of this study is to develop and characterise biobased, catalyst-free vitrimer epoxy systems and to evaluate their suitability as a sustainable matrix for natural fibre-reinforced composites by benchmarking their performance against a conventional epoxy system. Furthermore, this study aims to investigate the influence of vitrimer content on resin performance, particularly chemical recyclability, offering a novel and practical method for converting conventional epoxies into recyclable systems.

## 2. Materials and Methods

### 2.1. Materials

Vanillin (Reagent Plus, 99%), ethanol, methanol, dimethyl sulfoxide (DMSO), tetrahydrofuran (THF), dimethylformamide (DMF), dichloromethane (DCM), chloroform (CHCl_3_), toluene, sodium hydroxide (NaOH), and hydrochloric acid (HCl) were purchased from Sigma Aldrich. All chemicals were used without further purification.

Araldite^®^ LY 1564 epoxy resin and Aradur^®^ 3487 hardener were provided by Huntsman Corporation, Basel, Switzerland. This epoxy resin system is used as a reference resin and is named ER.

Woven flax fibre ampliTex™ 5040 (300 g/m^2^, twill 2/2) from Bcomp Ltd., Fribourg, Switzerland was purchased from Lange + Ritter GmbH, Gerlingen, Germany.

### 2.2. Preparation of Vitrimer Epoxy Resin (VER) and Vitrimer Epoxy Flax Fibre-Reinforced Composites (VER-FFRC)

To prepare vanillin-based vitrimer hardener (v-hardener), vanillin (10.0 g) was dissolved in ethanol (30.0 mL), and Aradur^®^ 3487 hardener (20.0 g) was slowly added to the vanillin solution at room temperature. The ratio of vanillin to Aradur hardener is 1:2. The mixture was stirred and left in the fume hood at room temperature for 5 h to evaporate the ethanol solvent. The remaining ethanol solvent and water by-product produced from the imine condensation between aldehyde (in vanillin) and primary amine (in Aradur^®^ 3487 hardener) were removed in a rotary evaporator. The obtained orange viscous oil is named v-hardener, containing dynamic imine bonds.

Two vanillin-based vitrimer epoxy resins (VER1-1 and VER1-2) were used as polymer matrices to fabricate the flax fibre-reinforced composites, in which VER1-1 contains a weight ratio of v-hardener to epoxy of 1:1, and VER1-2 contains a weight ratio of v-hardener to epoxy of 1:2. The detailed feed composition of the vitrimer epoxy flax composites (VER1-1-FFRC and VER1-2-FFRC) is shown in Table 1.

**Figure 1:**
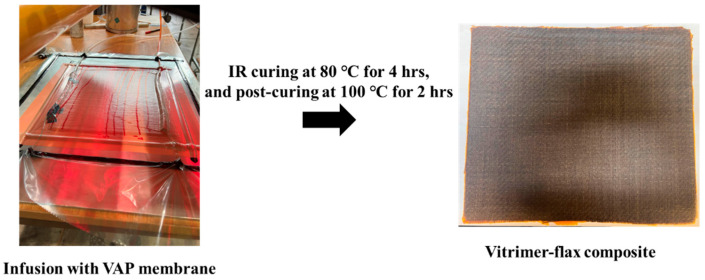
VARI setup for the fabrication of vitrimer epoxy flax fibre-reinforced composites.

Vitrimer epoxy flax fibre-reinforced composite samples (VER1-1-FFRC and VER1-2-FFRC) were fabricated with three layers of flax fibre fabric sheet, each measuring 300 mm × 350 mm. A metal plate was used as a flat tooling surface. The composites were fabricated using the VARI technique. The semi-permeable VAP^®^ membrane, sourced from Composyst GmbH (Landsberg am Lech, Germany), facilitates the degassing of trapped air and any gaseous by-products that may be generated during the vitrimer reaction. To enhance easy release from the tooling, release agent Zyvax^®^ TakeOff™, provided by Chem-Trend L.P., Howell, MI, USA, was applied. Peel plies were positioned on both sides of the FFRC plates. The entire setup assembly was initially heated to 40 °C before and during the infusion process. Following this, the temperature was gradually increased to 80 °C for curing over 4 h and subsequently to 100 °C for post-curing, which lasts for 2 h using an infrared (IR) heat lamp, as illustrated in Figure 1.

### 2.3. Characterisation

#### 2.3.1. Chemical Characterisation

Fourier transform infrared (FTIR) spectra were recorded using a Nicolet Diamond 5700 FTIR spectrometer (ThermoFisher, Waltham, MA, USA). Each spectrum was collected in a 4000 cm^−1^–400 cm^−1^ spectral range, with 32 scans at a 4 cm^−1^ resolution.

#### 2.3.2. Rheological Characterisation

The viscosity of each resin was measured using a rheometer from Anton Paar GmbH, Graz, Austria, with parallel plate geometry. The experiment involved scanning the shear rate between 0.1 s^−1^ and 100 s^−1^, recording the viscosity of the sample in response to varying shear rates. To measure the viscosity over time, each sample was maintained isothermally at 40 °C with a shear rate of 1 s^−1^, and the viscosity was recorded for 30 min.

#### 2.3.3. Morphological Characterisation

The fracture surfaces of the vitrimer epoxy flax fibre-reinforced composites were examined by using scanning electron microscopy (SEM) to investigate the fibre-matrix interfacial adhesion and failure mechanism. Prior to imaging, all samples were sputter-coated with a thin layer of gold (in 15 s) to ensure conductivity and minimise charging effects during SEM analysis.

#### 2.3.4. Thermomechanical Characterisation

Thermal gravimetric analysis (TGA) for the vitrimer epoxy flax fibre-reinforced composites was performed on a TA instrument with a scanning rate of 10 °C/min in a range of 25–800 °C under a nitrogen atmosphere.

Differential scanning calorimetry (DSC) for the vitrimer epoxy flax fibre-reinforced composites was performed on a Netzsch DSC, Polyma 214. The sample (~10 mg) was sealed in an aluminium crucible and heated from 25 °C to 130 °C at a heating rate of 10 °C/min. The sample was then cooled from 130 °C to 0 °C at a heating rate of 10 °C/min to remove the thermal history. After cooling, the sample was gradually heated to 130 °C with a slower heating rate of 5 °C/min to record the glass transition temperature T_g_ value. All the DSC cycles were performed under a nitrogen atmosphere (60 mL/min flow rate).

Dynamic mechanical analysis (DMA) was measured on a Q800 DMA (TA instrument, New Castle, DE, USA), operating in a three-point bending fixture with sample dimensions of approximately 40 mm × 10 mm × 2 mm (length × width × thickness). The temperature ramp was from 25 to 180 °C with the heating rate at 3 °C/min, and an oscillation frequency of 1 Hz. The tan delta peak recorded from the DMA was used as the T_g_ value of the cured composite samples.

#### 2.3.5. Mechanical Characterisation

Flexural properties were determined with four-point bending tests according to [22]. They were conducted using a universal testing machine Inspekt Table 20-1 (Hegewald & Peschke GmbH, Nossen, Germany), with a maximum force of 20 kN. Samples were cut to 60 mm × 15 mm × 2 mm using a milling machine. These samples were placed on two supporting fins with a width of 45 mm, and the flexural force was applied by a stamp with a width of 15 mm and a testing speed of 2 mm/min. The reported values are the average of at least five valid results.

#### 2.3.6. Solvent Resistance

A solvent resistance test for the vitrimer epoxy flax composites was performed by immersing the cut samples, with dimensions of 40 × 10 × 2 mm^3^, into different solvents (water, ethanol, HCl 1.0 M, H_2_SO_4_ 1.0 M, and NaOH 1.0 M) at room temperature for 7 days. After the immersion, the solvent was removed, and the swollen samples were gently dried with a Kimwipe (Merck, Melbourne, Australia); they were then weighed (*w*_1_) and dried in a vacuum oven at 80 °C for 24 h. The final vacuum-dried sample was weighed (*w*_2_). The swelling ratio and gel content were calculated based on the following equations:

$$\mathrm{Swelling}\;\mathrm{ratio}=(\frac{w_{1}-w_{0}}{w_{0}})\times 100$$

$$\mathrm{Gel}\;\mathrm{content}\;(\%)=(\frac{w_{2}}{w_{0}})\times 100$$

where *w*_0_ is the initial weight of the dried sample, *w*_1_ is the weight of the dried sample after the test, and *w*_2_ is the final weight after vacuum drying. In addition, after the test, the vacuum-dried composite samples were evaluated for their thermomechanical properties through DMA.

#### 2.3.7. Chemical Recyclability

A chemical recycling test for the vitrimer epoxy flax composites was conducted with Aradur hardener at 90 °C for 3 days. The vitrimer epoxy matrix was dissolved in the hardener through a transamination mechanism. The flax fibre (FF) was removed from the solution, washed with ethanol and then water, and dried in the vacuum oven at 100 °C for 24 h. Both the dissolved matrix and the recovered FF were then reused to fabricate the recycled flax composites, which were subsequently examined for their performance via TGA and DMA.

## 3. Results and Discussion

### 3.1. Formation and Structural Polymerisation of Vanillin-Based Vitrimer Hardener (V-Hardener) and Vitrimer Epoxy Resin (VER)

**Figure 2:**
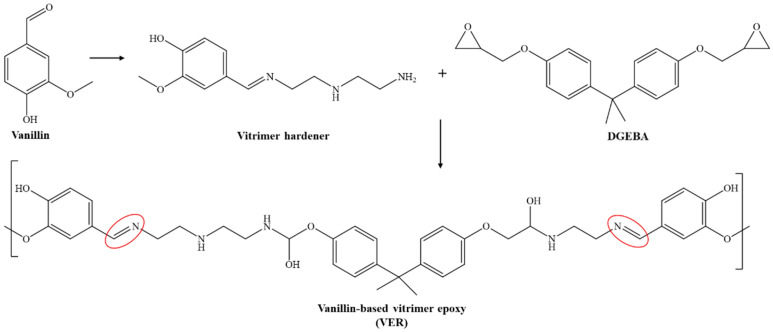
Proposed synthetic mechanism of vanillin-based vitrimer-epoxy resin (VER) (Imine bond C=N is shown in the red circle).

The overall synthetic mechanism of the vanillin-based vitrimer epoxy resin is shown in Figure 2. Briefly, vanillin reacted with the amine hardener (Aradur^®^ 3487) to form a vitrimer hardener, which was then added to the epoxy resin (Araldite^®^ LY 1564) to produce a vanillin-based vitrimer epoxy resin.

**Figure 3:**
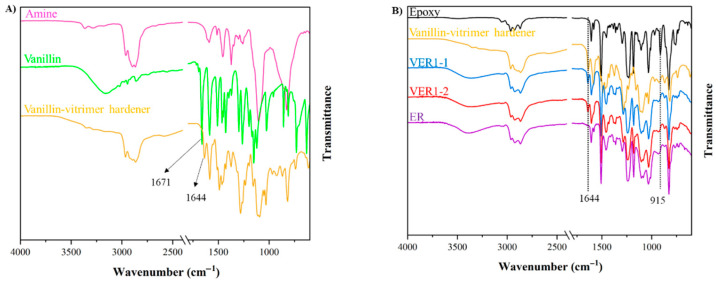
Normalised FTIR spectra of (**A**) amine hardener (Aradur^®^ 3487), vanillin, and vanillin-vitrimer hardener; and (**B**) vitrimer-epoxy resins (VER1-1, VER1-2) and epoxy resin reference (ER).

FTIR was performed to investigate the functional groups in the synthesised vanillin-based vitrimer hardener and the vitrimer epoxy resins. Figure 3A,B illustrate the functional groups in the vanillin-vitrimer hardener (v-hardener) and the comparison between the cured vitrimer-epoxy resins and the reference sample (ER), respectively. In Figure 3A, the characteristic peak observed at 1671 cm^−1^ is related to aromatic aldehyde (−C=O) in the vanillin molecule (green curve) [23]. This group disappeared in the v-hardener (orange curve) after the imine condensation reaction of the aldehyde group in vanillin with the amine in Aradur^®^ 3487 hardener. The new characteristic peak of the imine group C=N at 1644 cm^−1^ demonstrates the successful synthesis of v-hardener, which contains dynamic imine bonds [23,24]. Figure 3B presents the FTIR spectra of the cured VER1-1 and VER1-2 compared to the reference ER. The absorption peak at 915 cm^−1^, attributed to the epoxide functional group in the uncured epoxy (black curve), disappears after the curing process. A similar observation was previously reported by Yang and Wang [25], where the disappearance of the peak at 913 cm^−1^ and the appearance of a new easter peak at 1732 cm^−1^ confirmed epoxy group conversion through esterification. Furthermore, both VER1-1 and VER1-2 samples exhibit the imine C=N peak at 1644 cm^−1^, consistent with the incorporation of dynamic bonds [24], where this peak is absent in the ER reference sample.

### 3.2. Viscosity of the Vitrimer Epoxy Resin

**Figure 4:**
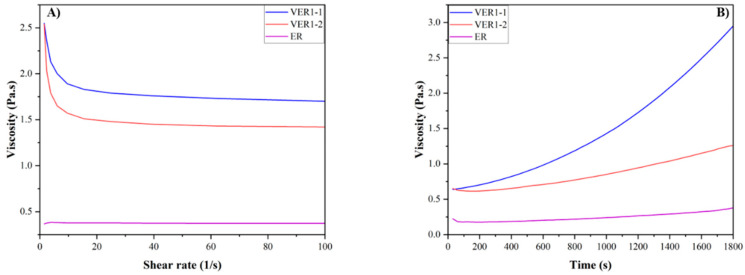
(**A**) Viscosity of different vitrimer epoxy resins (VER1-1 and VER1-2) and epoxy resin references (ER) in response to shear rate at room temperature, and (**B**) viscosity of different resins at 40 °C in response to time.

The viscosity of vitrimer epoxy resins is an important factor in their processing, particularly in the CFRC manufacturing process. Viscosity influences the resin’s ability to infiltrate the fibre matrix, ensuring uniform distribution and adhesion between the fibres and the resin. At elevated temperatures, the viscosity of the vitrimer resin typically decreases as the bond exchange reactions facilitate chain mobility, improving flow and wetting properties. This makes the resin suitable for processing techniques such as resin transfer moulding, vacuum-assisted resin infusion, and filament winding. The viscosity of vitrimer epoxy resins VER1-1 and VER1-2, as well as the reference epoxy resin ER, was investigated using an Anton Parr rheometer with the parallel plate configuration. The viscosity of the tested vitrimer epoxy resin samples both as a function of shear rate at room temperature and as a function of time at 40 °C is presented in Figure 4A and B, respectively.

As illustrated in Figure 4A, both VER1-1 and VER1-2 have a higher initial viscosity (2.5 Pa·s) compared to the reference epoxy resin (ER) (0.25 Pa·s). The high initial viscosity of VERs at room temperature is likely attributed to the presence of dynamic covalent bonds within the polymer network. While these dynamic bonds enable network rearrangement, they still limit polymer chain mobility at lower temperatures [26,27]. Moreover, the presence of dynamic bonds in addition to the static crosslinks results in a more rigid network, which leads to a higher viscosity, especially before the exchange reactions become active at elevated temperatures. Indeed, at 40 °C, the initial viscosity of both VER1-1 and VER1-2 dropped to 0.6 Pa·s (Figure 4B). The reduction in the viscosity of these vitrimer epoxies upon heating is primarily due to the dynamic bond exchange reactions, which allow for network rearrangement, lower crosslink density, and increased chain mobility. These changes result in a decrease in the vitrimer’s resistance to flow, making the resin more processable at higher temperatures over a limited time window. In other words, this reduction in viscosity at elevated temperatures is advantageous for the fabrication of CFRCs, as it allows for the easier processing and better infusion of the VER polymer matrix into the fibre. Figure 4B shows that all resins increase their viscosities over time, holding at 40 °C. Particularly, after 30 min, the viscosity of VER1-1 goes up to 3 Pa·s, whereas that of VER1-2 is 1 Pa·s and that of ER is 0.6 Pa·s. The higher viscosity of VER1-1 compared to VER1-2 and ER over time during exposure to heat is primarily due to the ongoing dynamic bond exchange reactions that lead to further crosslinking or network hardening. This increased crosslink density can make the material more rigid, resulting in higher viscosity as the resin becomes less flowable. Similarly to conventional epoxy resins, these vitrimer systems exhibit a defined processing window, i.e., a time frame during which the viscosity remains low enough for effective fibre impregnation and moulding. Based on the observed viscosity trends, VER1-1 has a narrower processing window (<30 min) than VER1-2 and ER. Defining and understanding this window is essential for processing optimisation and achieving consistent composite quality.

### 3.3. Fracture Morphology of the Vitrimer Flax Fibre-Reinforced Composites

**Figure 5:**
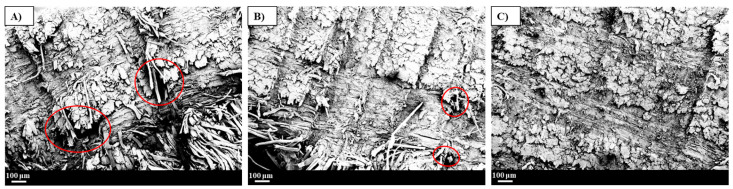
Cross-sectioned SEM images of vitrimer epoxy flax composites (**A**) VER1-1-FFRC, (**B**) VER1-2-FFRC, and (**C**) reference epoxy flax composite ER-FFRC. (Voids are shown in red circles).

SEM was performed to gain a clear understanding of the interfacial interaction between the flax fibre and the vitrimer epoxy resin of the VER1-1-FFRC, VER1-2-FFRC, and the reference ER-FFRC. The resulting SEM images are shown in Figure 5.

From the images, it is evident that the VER1-1-FFRC sample shows larger voids, whereas the VER1-2-FFRC and the ER-FFRC display fewer and smaller voids. This variation is likely due to the resin flow challenges encountered during the infusion process. Insufficient resin flow can lead to incomplete wetting of the fibres, resulting in the formation of air pockets or voids trapped within the composite structure. Such voids can significantly compromise the composite’s mechanical properties [28,29]. Therefore, it is anticipated that the ER-FFRC and VER1-2-FFRC may demonstrate better mechanical performance relative to the VER1-1-FFRC, owing to their reduced size and number of void contents. This hypothesis will be further examined through four-point flexural bending tests.

### 3.4. Thermomechanical Characteristics (TGA, DSC, and DMA)

**Figure 6:**
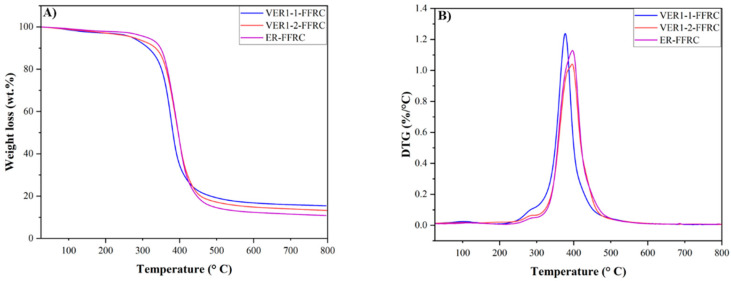
(**A**) Thermogravimetric (TG) and (**B**) derivative thermogravimetric (DTG) curves of vitrimer epoxy flax composites (VER1-1-FFRC and VER1-2-FFRC) and the reference epoxy resin flax composite (ER-FFRC).

The thermal stability of the vitrimer epoxy flax fibre-reinforced composites, VER1-1-FFRC and VER1-2-FFRC, and the conventional epoxy resin flax fibre-reinforced composite, ER-FFRC, was examined using the TGA under a nitrogen atmosphere. As shown in Figure 6 and Table 2, the initial degradation temperature (T_d5%_) of the VER1-1-FFRC (271.6 °C) is lower than that of the VER1-2-FFRC (274.5 °C) and the reference ER-FFRC (311.7 °C). These results indicate that the inclusion of vanillin-based vitrimer within the vitrimer epoxy system reduces the crosslink density, as detailed in Table 3, which in turn lowers the thermal stability of the crosslinked network. In other words, the initial degradation temperature decreases with the addition of vitrimer to the epoxy resin, a trend that is consistent with findings reported in previous studies [30,31,32].

Additionally, the VER1-1-FFRC and VER1-2-FFRC samples exhibit notable degradation temperatures (T_deg_) of 377.0 °C and 395.9 °C, respectively. These values are close to the thermal stability of the ER-FFRC, which shows a T_deg_ of 396.7 °C. The elevated degradation temperatures indicate that these VER-FFRCs possess adequate thermal stability for potential engineering applications. Comparable results have also been observed in recent research [31,32].

Additionally, the char residue remaining after combustion at 800 °C was observed to be highest in the VER1-1-FFRC, at 15.42 wt.%, followed by 12.44 wt.% for the VER1-2-FFRC and the lowest value of 10.82 wt.% for the ER-FFRC. The higher char residue observed in the VER1-1-FFRC and VER1-2-FFRC compared to the ER-FFRC can be attributed to the presence of benzene rings in vanillin and the C=N bond content in the vanillin-based vitrimer compound. This finding is consistent with previous reports [9,33]. The increased char residue in composite materials can possibly improve their flame resistance. When exposed to high temperatures, the char residue forms a stable, thermally insulating layer on the material’s surface. This layer acts as a barrier that reduces the rate of heat transfer into the material, effectively slowing down the decomposition and combustion processes. By preventing further breakdown of the material and limiting the spread of flames, the char residue helps enhance the overall flame resistance of the materials. In addition to the thermal insulation, this char layer can also act as a physical shield that prevents oxygen from reaching the underlying material, which is crucial for limiting fire propagation [33,34,35]. This mechanism is important for certain materials, especially composites, making them more resistant to fire and heat in applications like building construction, aerospace, and automotive industries.

**Figure 7:**
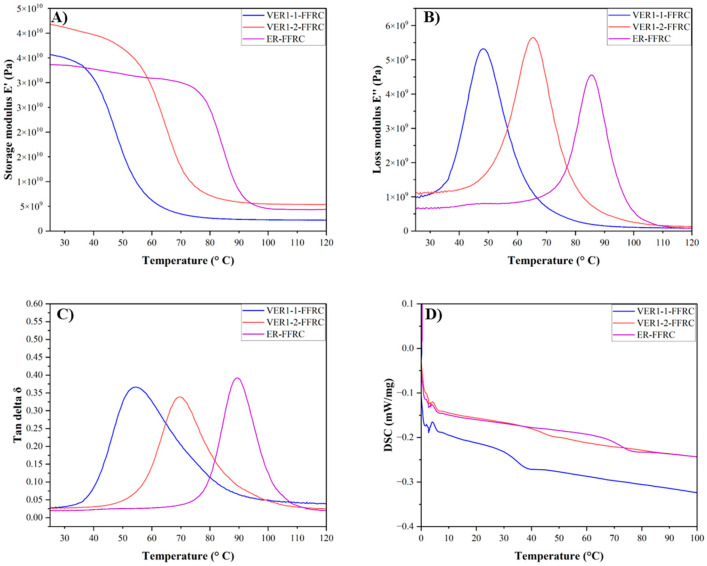
(**A**) Storage modulus ($E^{\prime }$), (**B**) loss modulus ($E^{\prime \prime }$), (**C**) tan delta curves from DMA, and (**D**) DSC second heating curves of vitrimer epoxy flax composites (VER1-1-FFRC and VER1-2-FFRC) and the reference epoxy resin flax composite (ER-FFRC).

The thermo-mechanical properties of the vitrimer epoxy composites were evaluated using a dynamic mechanical analyser (DMA). The primary dynamic properties—storage modulus, loss modulus, and tan delta—of the vitrimer epoxy composites (VER1-1-FFRC and VER1-2-FFRC), along with the reference composite ER-CFRC, are shown in Figure 7 and Table 3. The storage modulus ($E^{\prime }$) represents the material’s stiffness, reflecting the energy it retains during a loading cycle. The loss modulus ($E^{\prime \prime }$), or viscous modulus, indicates the material’s energy dissipation capability via internal molecular movements, characterising its damping behaviour. The ratio of $E^{\prime }$ to $E^{\prime \prime }$—the loss tangent (tan delta)—provides insight into this relationship. The maximum value of $E^{\prime \prime }/E^{\prime }$ (i.e., the peak tan delta) corresponds to the material’s glass transition temperature.

The glass transition temperature is also measured from the DSC analysis. As seen in Figure 7, the T_g_ decreases with the incorporation of vitrimer into the vitrimer epoxy polymer matrix. Specifically, the T_g_ values for the VER1-1-FFRC, VER1-2-FFRC, and ER-FFRC are 54.1 °C, 68.8 °C, and 83.4 °C, respectively. The observed decrease in T_g_ with the addition of vitrimer suggests that the crosslink density of the polymer network decreases. More specifically, the vitrimer networks, while still crosslinked, tend to have a lower crosslink density compared to traditional thermosets due to the reversible nature of the crosslinks (Table 3). As the crosslink density decreases, the polymer chains have greater mobility, leading to a lower T_g_. A lower T_g_ indicates that the material will transition from a rigid, glassy state to a more flexible, rubbery state at a lower temperature. This can be beneficial in applications where the material needs to exhibit more flexibility or impact resistance at lower temperatures, but it can also reduce the thermal stability of the material.

The crosslink density of these VER-FFRCs can be determined by DMA analysis using the theory of rubbery elasticity (Equation (1)) [9].

(1) $$\upsilon_{e}=\frac{E_{R}^{\prime }}{3RT}$$

where $E_{R}^{\prime }$ is the storage modulus at T_g_ + 30 °C, R is the gas constant (8.314 J·mol^−1^·K^−1^), and T is the absolute temperature at which $E_{R}^{\prime }$ is taken. As presented in Table 3, the crosslink density values for the vitrimer epoxy composites in this study (VER1-1-FFRC and VER1-2-FFRC) show that they have relatively lower crosslink densities compared to the ER-CFRC. Specifically, the VER1-1-FFRC has a crosslink density of 319.5 mol·m^−3^, which is lower than that of the VER1-2-FFRC (434.7 mol·m^−3^) and significantly lower than the reference ER-FFRC (442.9 mol·m^−3^).

### 3.5. Mechanical Characteristics (Flexural 4-Point Bending Test)

The mechanical flexural characteristics of the vitrimer epoxy flax composites (VER1-1-FFRC and VER1-2-FFRC) and the reference epoxy resin flax composite (ER-FFRC) were assessed by the four-point bending test. This test is commonly used to assess the flexural strength (i.e., the maximum stress the material can withstand before failure) and flexural stiffness (i.e., the material stiffness during bending, related to how much it deflects under a given load) of materials by applying a controlled load at two points while supporting the material at two other points. Therefore, comparing the results of the VER1-1-FFRC, VER1-2-FFRC, and ER-FFRC will give insight into how the vitrimer epoxy matrices (in the VER1-1-FFRC and VER1-2-FFRC) influence the mechanical properties of the flax fibre composites in terms of flexural performance. These are usually determined by the properties of the fibres and the resin, as well as the adhesion between those two. The different crosslink densities and the nature of the vitrimer system (which allows for reprocessing and potential self-healing) may impact these characteristics compared to the traditional epoxy resin flax composite (ER-FFRC).

The flexural modulus E_f_ is calculated via the following equation:

$$E_{f}=\frac{0.21L^{3}}{bh^{3}}\left(\frac{∆F}{∆s}\right)$$

where *L* is the support width, *b* is the width of the sample, and *h* is the thickness of the sample. ∆*s* describes the difference between deflections of the sample for edge fibre elongation (ε) at 0.0005 and 0.0025, and ∆*F* describes the difference between applied forces for *ε* at 0.0005 and 0.0025. Due to deflection values higher than 0.1 L, the standard requires a correction term in the equation for the flexural strength $\sigma_{f}$:

$$\sigma_{f}=\frac{FL}{bh^{2}}\left\{1\;+\;8.78{\left(\frac{s}{L}\right)}^{2}-7.04\left(\frac{sh}{L^{2}}\right)\right\}$$

**Figure 8:**
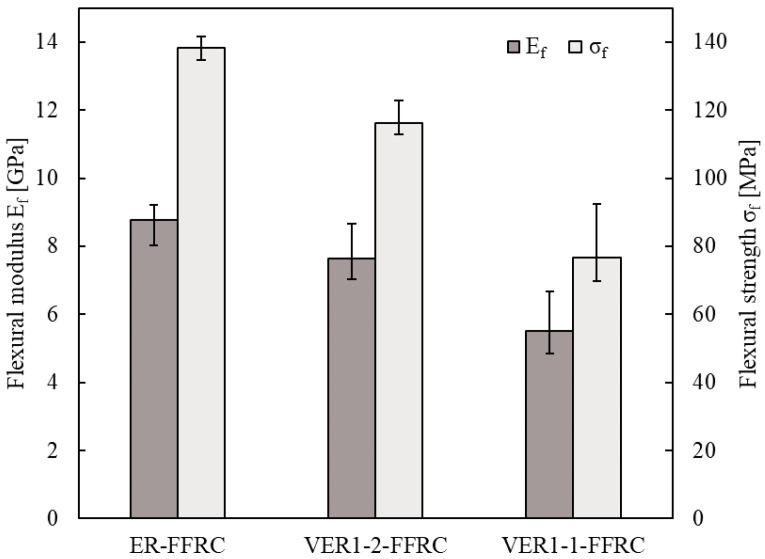
Four-point flexural modulus E_f_ (GPa) and flexural strength σ_f_ (MPa) of vitrimer epoxy flax composites (VER1-1-FFRC and VER1-2-FFRC) and the reference epoxy resin flax composite (ER-FFRC).

As illustrated in Figure 8, the VER1-1-FFRC exhibits a flexural modulus of 5.51 GPa, which is significantly lower than that obtained from the VER1-2-FFRC (7.63 GPa) and ER-FFRC (8.76 GPa). Similarly, the flexural strength of the VER1-1-FFRC, VER1-2-FFRC, and ER-FFRC is measured to be 76.7 MPa, 116.2 MPa, and 138.3 MPa, respectively. The reduction in the mechanical flexural characteristics of the vitrimer epoxy flax composite, especially of the VER1-1-FFRC, can be primarily attributed to its low crosslink density, which limits the stiffness and load-bearing capacity of the vitrimer network. Furthermore, the presence of dynamic covalent bonds in vitrimer may contribute to increased viscoelastic behaviour, potentially affecting the rigidity and resistance to deformation under bending loads [36,37] compared to traditional epoxy resins, which are typically more brittle and rigid [38]. With flexural strengths ranging from approximately 70 to 120 MPa and flexural moduli between 5 and 7 GPa, the VER1-1-FFRC and VER1-2-FFRC in this study demonstrate potential for use in semi-structural and structural automotive applications. For comparison, the typical flexural strength and modulus requirements for interior automotive components range from 37 to 76 MPa and 1.2 to 2.6 GPa, respectively [39,40]. These findings suggest that these vitrimer systems exceed the baseline requirements for such applications. However, it is important to note that mechanical performance can be enhanced by optimising the network design, such as increasing crosslink density or introducing molecular structures that restrict bond mobility. While such modification may comprise the ease of recyclability, they represent a promising direction for future research in balancing performance with sustainability.

### 3.6. Solvent Resistance Test

**Figure 9:**
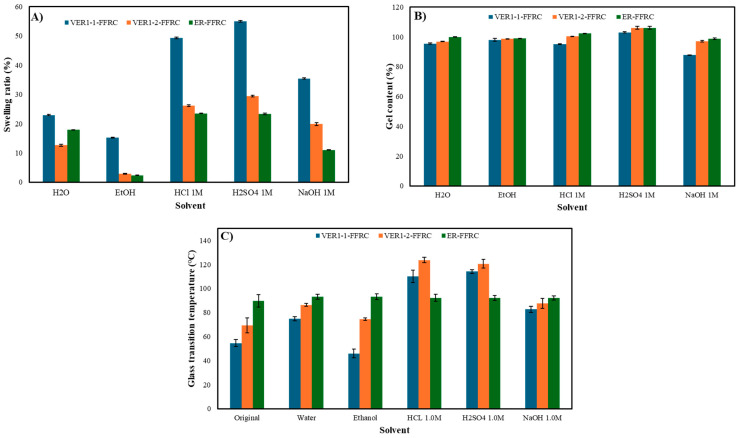
(**A**) Swelling ratio, (**B**) gel content, and (**C**) changes in the glass transition temperature after the solvent resistance test of vitrimer epoxy flax composites (VER1-1-FFRC and VER1-2-FFRC) and the reference epoxy resin flax composite (ER-FFRC).

Imines, due to the inherent properties of imine chemistry, are particularly susceptible to hydrolysis, particularly in the presence of moisture or under acidic or basic conditions. This can lead to a compromise in their structural integrity. In acidic environments, imine bonds within the vitrimer network may hydrolyse, breaking the imine linkage into amine and carbonyl components, which could potentially result in material degradation. To investigate this, a solvent resistance test was conducted by immersing cured composite samples in various solvents, including water (H_2_O), ethanol (EtOH), hydrochloric acid (HCl, 1.0 M), sulfuric acid (H_2_SO_4_, 1.0 M), and sodium hydroxide (NaOH, 1.0 M), at room temperature for 7 days. After immersion, the samples were removed, dried with Kimwipes, and weighed to assess any mass change due to swelling or dissolution. The samples were then placed in a vacuum oven and dried for 24 h. Following the drying process, dynamic mechanical analysis (DMA) was performed to determine the glass transition temperature, using the tan delta peak as an indicator of T_g_. The swelling ratios, gel content, and changes in the glass transition temperature for all samples after testing are presented in Figure 9A, B, and C, respectively.

As depicted in Figure 9A, the VER1-1-FFRC exhibited the highest swelling ratio across all solvents, with a particularly notable increase in acidic environments, where the swelling ratio exceeded 40%. This elevated swelling is likely due to the high vitrimer content within the vitrimer epoxy network, which lowers the material’s resistance to solvent interaction, particularly in acidic conditions where hydrolysis occurs. A similar behaviour was observed in a previous study, where cured vitrimers experienced significant swelling and degradation after 96 h of exposure to acidic solvents [31]. Additionally, the relatively low crosslink density of the VER1-1-FFRC (321.3 mol·m^−3^) contributes to its reduced solvent resistance compared to the other samples, the VER1-1-FFRC (433.5 mol·m^−3^) and ER-FFRC (440.1 mol·m^−3^).

The gel content of the three composite samples after immersion in various solvents is presented in Figure 9B. Gel content serves as an indicator of the degree of crosslinking in the polymer network, with higher values suggesting a greater extent of crosslinked polymer chains. A higher crosslink density generally correlates with enhanced mechanical performance, thermal stability, and chemical resistance [9,41]. As shown, the VER1-1-FFRC exhibits lower gel content across all tested solvents compared to the VER1-2-FFRC and the reference ER-FFRC. Specifically, the gel content of the VER1-1-FFRC ranges from approximately 88% to 95% (excluding H_2_SO_4_), whereas the VER1-2-FFRC displays values between 96% and 99% and the ER-FFRC maintains a consistent gel content of around 99%. These results suggest that the VER1-2-FFRC and ER-FFRC possess a denser crosslinked network, which is expected to contribute to more stable T_g_ following solvent exposure. Interestingly, all three samples show gel content values exceeding 100% after immersion in H_2_SO_4_. This anomaly may be attributed to sulphate salt or residual degradation products that remained on the samples after the drying process. This observation encourages further investigation into the effects of sulfuric acid exposure and sulphate attack on composite materials. The relationship between gel content, crosslink density, and T_g_ is further analysed through DMA, as shown in Figure 9C and Table 4.

DMA was also performed to assess the thermodynamic properties of the samples after the solvent resistance test. The T_g_ of the ER-FFRC remained stable, with a value around 90 °C. On the other hand, both the VER1-1-FFRC and VER1-2-FFRC were unstable in the testing solvents. Interestingly, the T_g_ of these vitrimer epoxy flax composites increased significantly after immersion in acidic solutions, rising from approximately 40 °C to 100 °C (Figure 9B). This increase in T_g_ after exposure to HCl 1.0 M and H_2_SO_4_ 1.0 M is likely due to changes in the polymer network and the dynamic imine bonds within the vitrimer system. While HCl may initially hydrolyse imine bonds, it may also promote the formation of more stable crosslinks within the vitrimer network. In some cases, HCl could trigger dynamic exchange reactions of the imine bonds, leading to network reorganisation and resulting in a more ordered, compact structure. This reorganisation would reduce the free volume and enhance molecular interactions, contributing to the observed increase in T_g_. An additional hypothesis is that the acidic environment may promote an increase in crosslink density, which would further explain the observed thermal behaviour (Table 4). However, current data do not provide direct evidence for increased crosslink density or improved mechanical properties as a result of acid treatment. These findings open up the possibility of incorporating this step as a secondary post-curing process during composite production. Future work will focus on investigating these changes in more depth, particularly through quantifying crosslink density and evaluating mechanical performance after acid treatment.

### 3.7. Chemical Recyclability in Aradur Hardener

**Figure 10:**
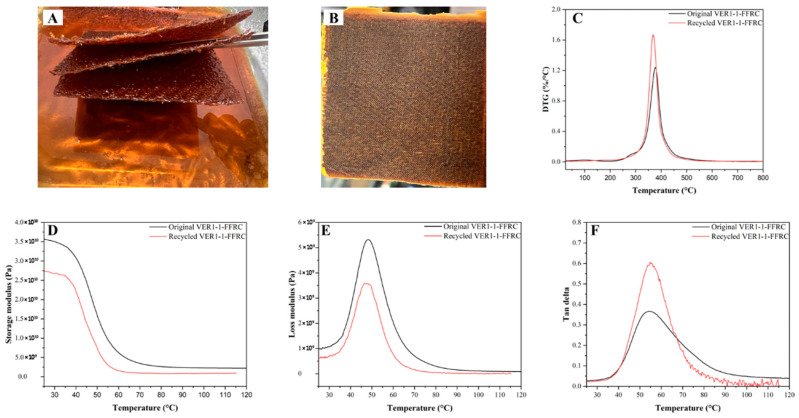
(**A**) Recovered flax fibres, (**B**) recycled vitrimer epoxy flax fibre composite (recycled VER1-1-FFRC), (**C**) derivative thermal degradation curves, (**D**) storage modulus curves, (**E**) loss modulus curves, and (**F**) tan delta curves of original and recycled materials after being recycled in Aradur hardener at 90 °C for 3 days.

Owing to the high vitrimer content in the polymer matrix, the VER1-1-FFRC was selected to investigate the recyclability of flax fibre composite materials. In brief, the VER1-1-FFRC plate was immersed in Aradur hardener, with the temperature maintained at 90 °C for 3 days. After this period, both flax fibres and the vitrimer epoxy resin (Figure 10A) were recovered and reused to fabricate a recycled vitrimer epoxy flax fibre composite, referred to as a recycled VER1-1-FFRC (Figure 10B).

The resulting recycled VER1-1-FFRC was subjected to TGA and DMA tests to evaluate its thermal and dynamic mechanical properties, respectively, in comparison with the original VER1-1-FFRC. Figure 10C–F present the derivative thermogravimetric (DTG) curves, storage modulus, loss modulus, and tan delta for the original and the recycled VER1-1-FFRC. As observed, the degradation temperature of the recycled sample closely matched that of the original VER1-1-FFRC, which is approximately 380 °C. This suggests that the thermal stability of the recycled composite was insignificantly affected by the chemical recycling approach. The preservation of thermal stability is a crucial factor in evaluating the effectiveness of recycling methods, as it suggests that the material retains its ability to withstand high temperatures, a key characteristic for practical applications. This finding aligns with previous studies [42] reporting vitrimer-based polymers with high thermal stability post-recycling due to their unique vitrimer network, which allows for the reversible exchange reactions without significant degradation of the overall polymer backbone.

However, a noticeable reduction in the storage modulus was observed following recycling. More specifically, the original VER1-1-FFRC exhibits a storage modulus of 3.49 × 10^10^ Pa, whereas the recycled VER1-1-FFRC shows a modulus of 2.68 × 10^10^ Pa at 30 °C. This decline in storage modulus can be related to the disruption of the crosslinking network during the recycling process, which involves the dissolution and reorganisation of the material’s molecular structure. During the recycling, the resin underwent partial depolymerisation and reformation, leading to a reduction in the overall density and order of the crosslinked network. As a result, the recycled material exhibits a lower stiffness. Such an observation is consistent with the findings of a previous study on vitrimer composites, where the recycling process was shown to cause a decrease in the storage modulus due to the increased heterogeneity of the polymer, which, in turn, reduces the crosslink density of the polymer network [43,44]. Interestingly, both the loss modulus and the tan delta peak, which corresponds to the glass transition temperature, exhibited only marginal changes following the recycling process. This indicates that the thermal response of the material remained relatively stable, suggesting that the recycling did not largely alter the intrinsic properties of the material’s glass transition behaviour. Similar trends have been reported in other vitrimer composites, where the glass transition temperature remained relatively unchanged even after multiple recycling cycles [44]. The glass transition temperature—a key indicator of the material’s flexibility and thermal performance at elevated temperatures—showed negligible change between the original and recycled composites. This suggests that while some mechanical properties (e.g., storage modulus) were affected, the overall thermal characteristics remained robust, highlighting the potential of vitrimer composites for recycling without compromising their performance in applications requiring thermal stability.

## 4. Conclusions

The integration of bio-based vitrimer epoxy resins with natural fibre reinforcement enhances the sustainability of fibre-reinforced composites by reducing the reliance on fossil fuels, improving recyclability, and offering biodegradable alternatives to synthetic materials. As research and development in this field continue, the potential for bio-based vitrimer epoxy natural fibre composites to become mainstream in high-performance applications looks increasingly promising. This work presented a fabrication of two vitrimer epoxy flax composites, named the VER1-1-FFRC and VER1-2-FFRC. It was found that the thermal stability of the VER1-1-FFRC (377.0 °C) and the VER1-2-FFRC (395.6 °C) was relatively high and comparable to the industry-grade epoxy flax composite, named the ER-CFRC (396.7 °C). However, due to the low crosslink density in the VER polymer matrix, the T_g_ of both the VER1-1-FFRC (54.1 °C) and VER1-2-FFRC (68.8 °C) was lower than that of the ER-FFRC (83.4 °C). Moreover, the mechanical performance of these VER-FFRCs is also reduced with the increased content of vitrimer in the vitrimer epoxy polymer matrix. The flexural strength of the VER1-1-FFRC was measured at 76.7 MPa, which is notably lower than that of both the VER1-2-FFRC (116.2 MPa) and the ER-FFRC (138.3 MPa). Despite these limitations, this work contributes to the broader understanding of the vitrimer–natural fibre systems and their potential in circular material design. Future efforts could focus on enhancing crosslink density or incorporating hybrid vitrimer systems to improve performance without compromising recyclability. Additionally, these composites may hold promising potential in some applications, such as automotive interior components, consumer goods, or construction panels, where sustainability and reprocessability are critical considerations. Further research into tailored formulations and scalable manufacturing methods could help bridge the gap between eco-friendliness and industrial applicability.

## Figures and Tables

**Table 1:** Feed compositions (in grams) of vanillin-based vitrimer hardener (v-hardener), vitrimer-epoxy resins (VER1-1 and VER1-2), and vitrimer-epoxy flax composites (VER1-1-FFRC, VER1-2-FFRC, and the reference ER-FFRC).

Samples	Vanillin (grams)	Aradur^®^ 3487 Hardener (grams)	v-Hardener (grams)	Araldite^®^ LY 1564 Epoxy (grams)	Curing Conditions
**v-hardener**	10	20	-	-	
**VER1-1**	-	-	10	10	40 °C before and during the infusion 80 °C curing 4 h 100 °C post-curing 2 h
**VER1-2**	-	-	10	20	
**VER1-1-FFRC**	-	-	100	100	
**VER1-2-FFRC**	-	-	75	150	
ER-FFRC (reference) *	-	34	-	100	
Fibre volume fraction for all flax composite samples is 30%					

* Epoxy/amine hardener weight ratio was based on the supplier datasheet (Huntsman).

**Table 2:** Thermal stability characteristics of VER-FFRC.

Sample	T_d5%_ (℃)	T_deg_ (℃)	Char Residue at 800 °C (%)
**VER1-1-FFRC**	271.6	377.0	15.42
**VER1-2-FFRC**	274.5	395.9	12.44
**ER-FFRC Reference**	311.7	396.7	10.82

T_d5%_: initial degradation temperature at 5% weight loss. T_deg_: degradation temperature.

**Table 3:** Dynamic mechanical characteristics of VER-FFRC.

Sample	T_g_^a^ (°C)	T_g_^b^ (°C)	$E_{R}^{\prime }$ (MPa)	υ_e_ (mol·m^−3^)
**VER1-1-FFRC**	54.1	34.4	2872.9	321.3
**VER1-2-FFRC**	68.8	42.7	4034.1	433.5
**ER-FFRC Reference**	83.4	71.2	4298.2	440.1

T_g_^a^ is the glass transition temperature as determined by the peak of the tan delta from DMA. T_g_^b^ is the glass transition temperature determined by DSC. $E_{R}^{\prime }$ is the storage modulus from the DMA at T_g_ + 30 °C. υ_e_: crosslinked density.

**Table 4:** Glass transition from tan delta peak and crosslinked density (υ_e_) estimated from the DMA of samples before and after the solvent resistance test.

Sample ID		T_g_ (°C)	υ_e_ (mol·m^−3^)
**VER1-1-FFRC**	Original	54.1 ± 2.07	319.5 ± 2.47
	Water	73.0 ± 2.14	233.2 ± 25.3
	Ethanol	47.7 ± 6.12	221.0 ± 1.41
	HCl 1.0 M	109.6 ± 1.82	580.2 ± 3.75
	H_2_SO_4_ 1.0 M	109.7 ± 4.80	616.7 ± 39.6
	NaOH 1.0 M	77.4 ± 6.43	215.1 ± 34.3
**VER1-2-FFRC**	Original	68.8 ± 1.89	434.7 ± 1.68
	Water	83.6 ± 2.79	369.6 ± 12.6
	Ethanol	71.9 ± 3.30	359.7 ± 4.78
	HCl 1.0 M	115.2 ± 11.7	433.9 ± 35.8
	H_2_SO_4_ 1.0 M	111.4 ± 12.6	401.3 ± 34.1
	NaOH 1.0 M	84.8 ± 5.17	463.0 ± 9.14
**ER-FFRC**	Original	83.4 ± 7.11	442.9 ± 3.87
	Water	87.2 ± 7.80	383.4 ± 17.6
	Ethanol	86.7 ± 8.85	456.8 ± 8.93
	HCl 1.0 M	87.7 ± 6.70	485.6 ± 37.5
	H_2_SO_4_ 1.0 M	87.8 ± 7.37	420.6 ± 11.6
	NaOH 1.0 M	87.9 ± 6.08	376.3 ± 15.5

## Data Availability

The original contributions presented in this study are included in the article. Further inquiries can be directed to the corresponding authors.

## References

[1] Dong K., Tang S., Zhao D., Pang Y., Zhao C. (2024). Vanillin-derived bio-based epoxy resins containing dual dynamic Schiff base and disulfide bonds with reprocessability and degradability. Polym. Degrad. Stab..

[2] Verma S., Gangil B., Ranakoti L., Verma J., Verma A., Jain N., Rangappa S.M., Siengchin S., Matykiewicz D. (2024). 4—Study of physical, thermal, and mechanical properties of thermosetting polymer composites. Dynamic Mechanical and Creep-Recovery Behavior of Polymer-Based Composites.

[3] Gangil B., Kumar S., Tejyan S., Ranakoti L., Verma S., Verma A., Jain N., Rangappa S.M., Siengchin S., Matykiewicz D. (2024). 2—Introduction to thermosetting polymer composites: Applications, advantages, and drawbacks. Dynamic Mechanical and Creep-Recovery Behavior of Polymer-Based Composites.

[4] Ren Y., Hu H., Lin Z., Cao C., Liu H., Li X., Yao H. (2024). A novel method of reclaiming high-value carbon fiber from waste epoxy composite via molten salt thermal treatment. Chem. Eng. J..

[5] Khan F.M., Shah A.H., Wang S., Mehmood S., Wang J., Liu W., Xu X. (2022). A comprehensive review on epoxy biocomposites based on natural fibers and bio-fillers: Challenges, recent developments and applications. Adv. Fiber Mater..

[6] Monteserin C., Blanco M., Uranga N., Sanchez J., Laza J.M., Vilas J.L., Aranzabe E. (2023). Sustainable biobased epoxy thermosets with covalent dynamic imine bonds for green composite development. Polymer.

[7] Chen C., Yang Y., Zhou Y., Xue C., Chen X., Wu H., Sui L., Li X. (2020). Comparative analysis of natural fiber reinforced polymer and carbon fiber reinforced polymer in strengthening of reinforced concrete beams. J. Clean. Prod..

[8] Xu Y., Dai S., Zhang H., Bi L., Jiang J., Chen Y. (2021). Reprocessable, Self-Adhesive, and Recyclable Carbon Fiber-Reinforced Composites Using a Catalyst-Free Self-Healing Bio-Based Vitrimer Matrix. ACS Sustain. Chem. Eng..

[9] Tran H., Radjef R., Nikzad M., Bjekovic R., Fox B. (2024). A vanillin-based vitrimer matrix for recyclable and sustainable carbon fibre-reinforced composites. J. Clean. Prod..

[10] Zhou X., Shen M., Fu F., Li Q., Liu H., Song Z. (2024). High strength, self-healing and hydrophobic fully bio-based polybenzoxazine reinforced pine oleoresin-based vitrimer and its application in carbon fiber reinforced polymers. Chem. Eng. J..

[11] Hong J., Hong Y., Jeong J., Oh D., Goh M. (2023). Robust Biobased Vitrimers and Its Application to Closed-Loop Recyclable Carbon Fiber-Reinforced Composites. ACS Sustain. Chem. Eng..

[12] Xu Y., Zhang H., Dai S., Xu S., Wang J., Bi L., Jiang J., Chen Y. (2022). Hyperbranched polyester catalyzed self-healing bio-based vitrimer for closed-loop recyclable carbon fiber-reinforced polymers. Compos. Sci. Technol..

[13] Tran H.T., Nisha S.S., Radjef R., Nikzad M., Bjekovic R., Fox B. (2024). Recyclable and Biobased Vitrimers for Carbon Fibre-Reinforced Composites—A Review. Polymers.

[14] Debsharma T., Engelen S., De Baere I., Van Paepegem W., Du Prez F. (2023). Resorcinol-Derived Vitrimers and Their Flax Fiber-Reinforced Composites Based on Fast Siloxane Exchange. Macromol. Rapid Commun..

[15] Fei M., Liu W., Shao L., Cao Y., Bliss B.J., Zhao B., Zhang J. (2024). Hemp fiber reinforced dual dynamic network vitrimer biocomposites with direct incorporation of amino silane. Chem. Eng. J..

[16] Martinez P., Nutt S. (2024). Flax–reinforced vitrimer epoxy composites produced via RTM. J. Compos. Sci..

[17] Rohewal S.S., Yu Z., Kearney L.T., Toomey M.D., Ghossein H.K., Naskar A.K. (2024). Flax fiber-reinforced fatty acid vitrimer biocomposite with enhanced chemical recyclability. MRS Commun..

[18] Luo C., Yang W., Qi W., Chen Z., Lin J., Bian X., He S. (2022). Cost-efficient and recyclable epoxy vitrimer composite with low initial viscosity based on exchangeable disulfide crosslinks. Polym. Test..

[19] Obadia M.M., Jourdain A., Cassagnau P., Montarnal D., Drockenmuller E. (2017). Tuning the viscosity profile of ionic vitrimers incorporating 1, 2, 3-triazolium cross-links. Adv. Funct. Mater..

[20] Formon G.J., Storch S., Delplanque A.Y.G., Bresson B., Van Zee N.J., Nicolaÿ R. (2023). Overcoming the tradeoff between processability and mechanical performance of elastomeric vitrimers. Adv. Funct. Mater..

[21] Schenk V., Labastie K., Destarac M., Olivier P., Guerre M. (2022). Vitrimer composites: Current status and future challenges. Mater. Adv..

[22] (2011). Fibre-Reinforced Plastic Composites—Determination of Flexural Properties.

[23] Rashid M.A., Zhu S., Jiang Q., Wei Y., Liu W. (2022). Developing easy processable, recyclable, and self-healable biobased epoxy resin through dynamic covalent imine bonds. ACS Appl. Polym. Mater..

[24] Liu J., Liu X., Cui X., Qin J., Shi M., Wang D., Liang L., Yang C. (2023). Imine-Containing Epoxy Vitrimer Cured by Active Ester: Properties and Theoretical Analysis. ACS Appl. Polym. Mater..

[25] Yang H., Wang D. (2024). Comparing Surface and Bulk Curing Processes of an Epoxy Vitrimer. ACS Appl. Mater. Interfaces.

[26] Soman B., Evans C.M. (2021). Effect of precise linker length, bond density, and broad temperature window on the rheological properties of ethylene vitrimers. Soft Matter.

[27] Taplan C., Guerre M., Winne J.M., Du Prez F.E. (2020). Fast processing of highly crosslinked, low-viscosity vitrimers. Mater. Horiz..

[28] Klosterman D., Browning C., Hakim I., Lach K. (2021). Investigation of various techniques for controlled void formation in fiberglass/epoxy composites. J. Compos. Mater..

[29] Saeed K., McIlhagger A., Harkin-Jones E., McGarrigle C., Dixon D., Shar M.A., McMillan A., Archer E. (2022). Characterization of continuous carbon fibre reinforced 3D printed polymer composites with varying fibre volume fractions. Compos. Struct..

[30] Liu T., Hao C., Zhang S., Yang X., Wang L., Han J., Li Y., Xin J., Zhang J. (2018). A Self-Healable High Glass Transition Temperature Bioepoxy Material Based on Vitrimer Chemistry. Macromolecules.

[31] Memon H., Wei Y., Zhang L., Jiang Q., Liu W. (2020). An imine-containing epoxy vitrimer with versatile recyclability and its application in fully recyclable carbon fiber reinforced composites. Compos. Sci. Technol..

[32] Memon H., Wei Y., Zhu C. (2021). Correlating the thermomechanical properties of a novel bio-based epoxy vitrimer with its crosslink density. Mater. Today Commun..

[33] Zamani P., Zabihi O., Ahmadi M., Mahmoodi R., Kannangara T., Joseph P., Naebe M. (2021). Biobased Carbon Fiber Composites with Enhanced Flame Retardancy: A Cradle-to-Cradle Approach. ACS Sustain. Chem. Eng..

[34] Zamani P., Zabihi O., Ahmadi M., Mahmoodi R., Naebe M. (2024). The Quest for Sustainable Composites: Developing High-Performance, Flame-Retardant, and Recyclable Carbon Fiber-Reinforced Epoxy Vitrimer Composites. ACS Appl. Polym. Mater..

[35] Zamani P., Zabihi O., Ahmadi M., Zamani M.R., Zohuriaan-Mehr M.J., Kannangara T., Joseph P., Naebe M. (2024). Assessing sustainability and green chemistry in synthesis of a Vanillin-based vitrimer at scale: Enabling sustainable manufacturing of recyclable carbon fiber composites. Compos. Part A Appl. Sci. Manuf..

[36] Meng F., Saed M.O., Terentjev E.M. (2022). Rheology of vitrimers. Nat. Commun..

[37] Xia J., de la Cruz M.O. (2024). Effect of molecular structure on the dynamics and viscoelasticity of vitrimers. Polymer.

[38] Chong K.L., Lai J.C., Rahman R.A., Adrus N., Al-Saffar Z.H., Hassan A., Lim T.H., Wahit M.U. (2022). A review on recent approaches to sustainable bio-based epoxy vitrimer from epoxidized vegetable oils. Ind. Crops Prod..

[39] Javanshour F., Prapavesis A., Pärnänen T., Orell O., Lessa Belone M.C., Layek R.K., Kanerva M., Kallio P., Van Vuure A.W., Sarlin E. (2021). Modulating impact resistance of flax epoxy composites with thermoplastic interfacial toughening. Compos. Part A Appl. Sci. Manuf..

[40] Malik K., Ahmad F., Dawood M.S.I.S., Islam M.S., Ali S., Raza A., Shahed C.A. (2024). Mechanical property enhancement of graphene-kenaf-epoxy multiphase composites for automotive applications. Compos. Part A Appl. Sci. Manuf..

[41] Jing F., Zhao R., Li C., Xi Z., Wang Q., Xie H. (2022). Influence of the epoxy/acid stoichiometry on the cure behavior and mechanical properties of epoxy vitrimers. Molecules.

[42] Tian P.-X., Li Y.-D., Hu Z., Zeng J.-B. (2024). Fire-resistant and high-performance epoxy vitrimers for fully recyclable carbon fiber-reinforced composites. Mater. Today Chem..

[43] Hubbard A.M., Ren Y., Sarvestani A., Konkolewicz D., Picu C.R., Roy A.K., Varshney V., Nepal D. (2022). Recyclability of Vitrimer materials: Impact of catalyst and processing conditions. ACS Omega.

[44] Mao H.-I., Hu J.-Y., Shiu J.-W., Rwei S.-P., Chen C.-W. (2023). Sustainability and repeatedly recycled epoxy-based vitrimer electromagnetic shielding composite material. Polym. Test..

